# Implementation of a malaria sentinel surveillance system in Togo: a pilot study

**DOI:** 10.1186/s12936-020-03399-y

**Published:** 2020-09-09

**Authors:** Anne Thomas, Tchaa A. Bakai, Tinah Atcha-Oubou, Tchassama Tchadjobo, Nicolas Voirin

**Affiliations:** 1 Epidemiology and Modelling in Infectious Diseases (EPIMOD), Dompierre-sur-Veyle, France; 2 Programme National de Lutte Contre Le Paludisme (PNLP), Lomé, Togo

**Keywords:** Surveillance system, Sentinel sites, Malaria, National malaria control programme, Togo

## Abstract

**Background:**

In Togo, the National Malaria Control Programme, in collaboration with the Global Fund to Fight AIDS, Tuberculosis and Malaria, has implemented a pilot study for malaria sentinel surveillance since 2017, which consists of collecting information in real time and analysing this information for decision-making. The first 20 months of malaria morbidity and mortality trends, and malaria case management in health facilities included in the surveillance were assessed.

**Methods:**

Since July 2017, 16 health facilities called sentinel sites, 4 hospitals and 12 peripheral care units located in 2 epidemiologically different health regions, have provided weekly data on malaria morbidity and mortality for the following 3 target groups: < 5-years-old children, ≥ 5-years-old children and adults, and pregnant women. Data from week 29 in 2017 to week 13 in 2019 were analysed.

**Results:**

Each sentinel site provided complete data and the median time to data entry was 4 days. The number of confirmed malaria cases increased during the rainy seasons both in children under 5 years old and in children over 5 years old and adults. Malaria-related deaths occurred mainly in children under 5 years old and increased during the rainy seasons. The mean percentage of tested cases for malaria among suspected malaria cases was 99.0%. The mean percentage of uncomplicated malaria cases handled in accordance with national guidelines was 99.4%. The mean percentage of severe malaria cases detected in peripheral care units that were referred to a hospital was 100.0%. Rapid diagnostic tests and artemisinin-based combination therapies were out of stock several times, mainly at the beginning and end of the year. No hospital was out of stock of injectable artesunate or injectable artemether.

**Conclusions:**

These indicators showed good management of malaria cases in the sentinel sites. Real-time availability of data requires a good follow-up of data entry on the online platform. The management of input stocks and the promptness of data need to be improved to meet the objectives of this malaria sentinel surveillance system.

## Background

In 2017, the World Health Organization (WHO) African Region still had the highest number of malaria cases (92%) and malaria deaths (93%). *Plasmodium falciparum* is the predominant malaria parasite in the WHO African Region and was responsible for 99.7% of malaria cases in this region in 2017 [1].

In Togo, the estimated number of malaria cases was 2.9 million and the estimated number of malaria deaths was 5,341 in 2017 [1]. In 2016, uncomplicated malaria was the first cause of outpatient consultations (41.7%) and severe malaria was the first cause of hospitalizations (20.5%) and the ninth cause of hospital mortality (3.8%) in Togo [2]. Malaria deaths are probably underestimated as 60.3% of severe malaria cases that were referred did not go to hospital in 2017 [3]. One of the most vulnerable groups affected by malaria are children under 5 years old [4], accounting for 35.4% of uncomplicated malaria cases, 58.4% of hospitalized severe malaria cases and 69.7% of malaria deaths in 2017 in Togo [3].

The current Togolese national policy is based on free and universal access to diagnosis and treatment of malaria in public health facilities and at community level by community health workers [5]. Over the past several years, the National Malaria Control Programme has adopted large-scale interventions to prevent malaria in different target groups, such as: (i) distribution of long-lasting insecticidal nets in routine medical visits among pregnant women and children under 1 year old; (ii) mass net distribution every 3 years to the entire country; (iii) distribution of seasonal malaria chemoprevention from July to September in children aged 3–59 months in 3 health regions (Centrale, Kara, Savanes); and, (iv) intermittent preventive treatment during pregnancy.

According to the Global Technical Strategy for Malaria 2016–2030 report [6], surveillance is a pillar of malaria control and elimination programmes (pillar 3 of the report). Monitoring malaria dynamics over time and space allows active and appropriate measures to be taken based on the data collected. This information is crucial to implement sustainable and efficient interventions [7, 8]. In Togo, a routine malaria surveillance system has collected data monthly and by district since 1956. Since 2017, a pilot study has been implemented to collect malaria data on a more accurate scale; each week at the level of health facilities. The medium and long-term objectives of this malaria sentinel surveillance system are to provide accurate and timely data on morbidity and mortality trends, to monitor occurrence and progression of epidemic episodes, to facilitate rapid responses at medical and programmatic levels, to evaluate malaria control interventions, and to monitor progress towards malaria elimination [9].

Here the Togolese malaria sentinel surveillance system aiming to collect and report data was described. The first 20 months of malaria morbidity and mortality trends, and malaria case management in health facilities included in the surveillance were assessed.

## Methods

### Setting

Togo is a country in West Africa, bordered by Ghana, Burkina Faso, Benin, and the Bight of Benin. Togo is one of the smallest countries in Africa, with a total land mass of 56,785 sq km [10]. According to World Bank data, Togo’s population was estimated at 7.9 million in 2018 [11]. Population density is highest in the south where the capital Lomé is located, although the inhabitants reside mainly in rural areas. Togo is composed of 6 health regions (from north to south: Savanes, Kara, Centrale, Plateaux, Maritime, Lomé-commune), and 40 health districts. In 2016, Togo had 1,224 health facilities, including 504 private health facilities, and 261 pharmaceutical facilities [2].

### Selection of sentinel sites

**Fig. 1:**
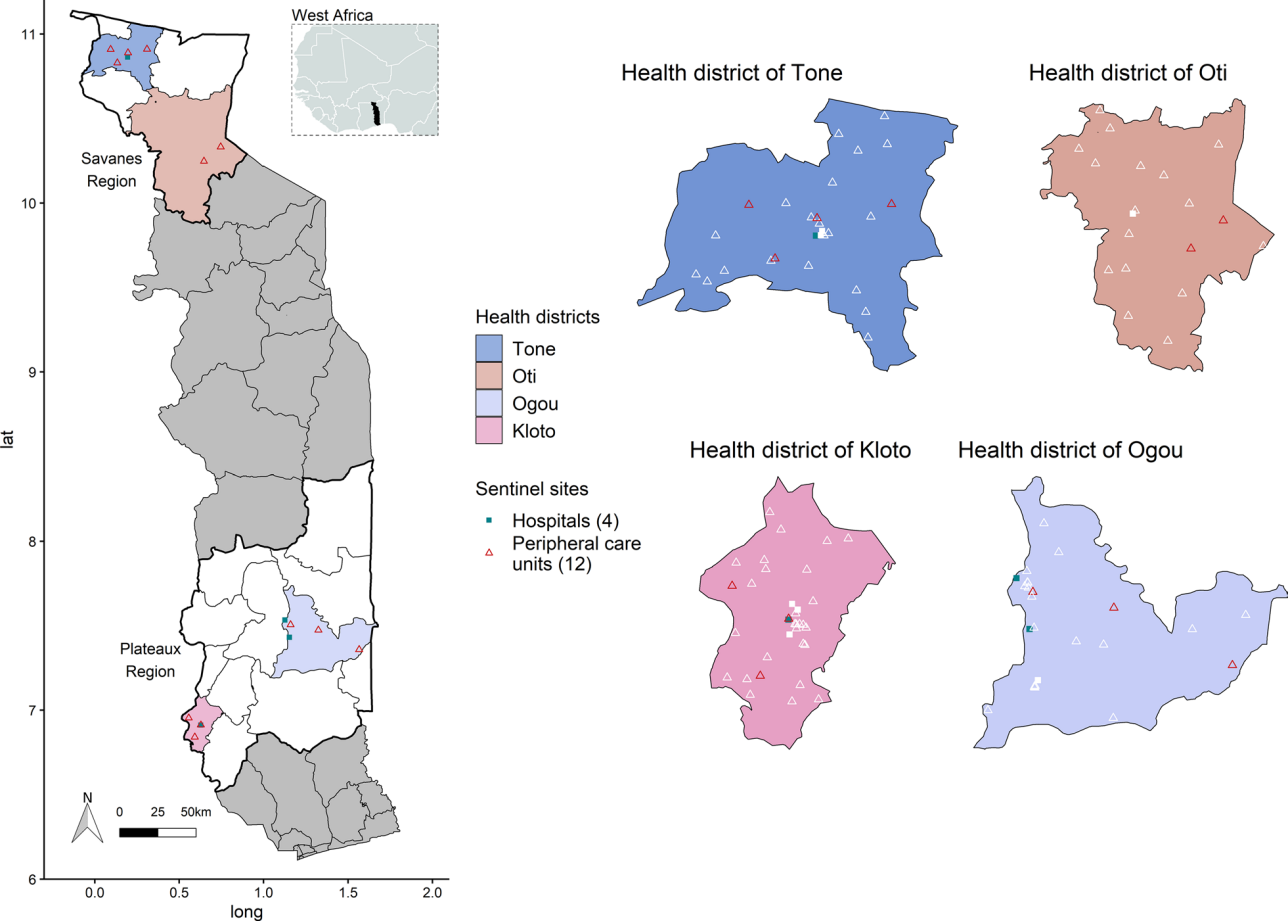
Location of the 16 sentinel sites in the four health districts included in the pilot malaria sentinel surveillance system, Togo. Squares represent hospitals (sentinel sites in green and all other hospitals in white) and triangles represent peripheral care units (sentinel sites in red and all other peripheral care units in white)

Since July 2017, the National Malaria Control Programme, in collaboration with the Global Fund to Fight AIDS, Tuberculosis and Malaria, has implemented a pilot study for malaria sentinel surveillance in 2 epidemiologically different health regions (the Savanes and Plateaux regions) and 4 health districts: 2 districts in each region (the Tone, Oti, Ogou, Kloto districts). Togo has a tropical climate characterized by a difference between north and south. The Savanes region is located in the north, where there is a long rainy season from May to October, while the Plateaux region is located in the south where there are 2 rainy seasons from March to July and in September and October [2]. In addition, the Savanes region benefits from specific malaria control activities such as the distribution of seasonal malaria chemoprevention in children under 5 years old, while the Plateaux region does not. Health districts and health facilities were also selected based on proven experience of district managers, site managers and health workers in malaria surveillance activities, and health facility accessibility. At the beginning of this project, 17 health facilities, then called sentinel sites, were included. However, a faith-based hospital in the Oti district, not accustomed to working with the National Malaria Control Programme, did not provide any data and was therefore excluded from data analysis. Of the 16 sentinel sites, there are 4 hospitals and 12 peripheral care units. Figure 1 represents the project area and the location of the sentinel sites in Togo.

### Malaria case management in sentinel sites

Malaria case management in sentinel sites complies with the following national recommendations [5]:

1. All suspected cases of malaria should be tested by a diagnostic test (rapid diagnostic test (RDT) or microscopy).
2. All uncomplicated malaria cases should be treated with artemisinin-based combination therapies (ACT). Artemether-lumefantrine is recommended as first-line treatment, and artesunate-amodiaquine is recommended as a second-line treatment. Quinine is recommended for pregnant women in their first trimester. Prescribing and providing, free of charge, the recommended anti-malarial treatment or prescribing only the recommended anti-malarial treatment to the patient is considered to be in accordance with national recommendations.
3. All severe malaria cases detected in peripheral care units should be referred to a hospital. Injectable artesunate is recommended as a first-line treatment and injectable artemether is recommended as a second-line treatment. These recommendations apply to the pre-transfer as well. However, artesunate in suppository form is recommended as a first-line pre-transfer treatment in children under 5 years old.

### Definition of indicators

A suspected malaria case was defined as a person presenting in a sentinel site with a temperature ≥ 37.5 °C or a history of fever in the previous 24 h. A tested case for malaria was defined as a suspected malaria case that received a diagnostic test, either by microscopy or by RDT. A confirmed malaria case was defined as tested case with a positive diagnostic test. According to WHO guidelines, an uncomplicated malaria case was defined as a confirmed malaria case with no features of severe malaria. A severe malaria case was defined as a confirmed malaria case with one or more of the following criteria: impaired consciousness, prostration, multiple convulsions, acidosis, hypoglycaemia, severe malarial anaemia, renal impairment, jaundice, pulmonary oedema, significant bleeding, shock, hyperparasitaemia [12]. A malaria-related death was defined as a hospitalized confirmed malaria case that dies. Only hospitals recorded malaria-related deaths. Data completeness was defined as the percentage of entered forms on the online platform among the number of expected forms per week. Data promptness was defined as the percentage of entered forms on the online platform before Thursday of the following week among the number of expected forms per week. Stock shortage was defined as the number of days of disruption of RDTs, ACT, or artesunate or artemether injections in each sentinel site a given week.

### Data collection

This malaria sentinel surveillance system provided weekly data on malaria morbidity and mortality for the following 3 target groups: < 5-years-old children, ≥ 5-years-old children and adults (excluding pregnant women), and pregnant women. Primary data sources were consultation registers that included items such as temperature, symptoms, diagnosis, requested medical examinations, results and treatments. A standardized paper form was developed for weekly data collection in each sentinel site allowing the surveillance to start in July 2017.

### Data entry and data validation

**Fig. 2:**
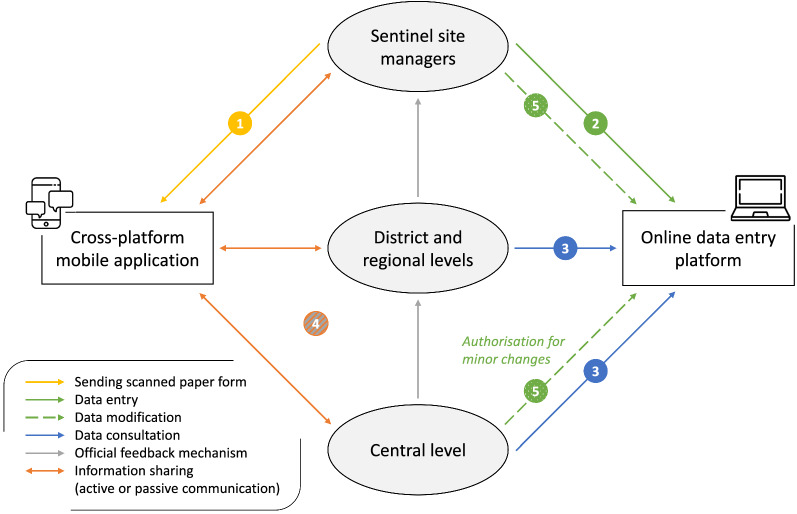
Flow chart of the reporting and feedback mechanisms in the pilot malaria sentinel surveillance system, Togo

An online data entry platform was set up and was operational in week 26, 2018. From this date, each site manager was able to enter the data prospectively through the online platform, while data prior to this date were entered retrospectively. Promptness was therefore calculated when the online data entry platform was set up. Input controls were integrated progressively into the online data entry platform to limit errors and aberrant data. Figure 2 describes the reporting and feedback mechanisms in the pilot malaria sentinel surveillance system. Each site manager shared the scanned paper forms on the cross-platform mobile application and entered the data on the online data entry platform. District, regional and central managers could consult the data entered. District managers checked the accuracy of data between the consultation registers and the paper forms, while regional and central managers mainly checked the accuracy of data between the paper forms and the data entered. Site managers were contacted when data were aberrant or not entered. Two feedback mechanisms could then be used: the official feedback mechanism (by the direct hierarchic level) or the feedback mechanism through the cross-platform mobile application for rapid information sharing to all stakeholders. Data were corrected after checking the paper forms or the consultation registers by site managers. For minor changes, central managers could correct the data after informing the relevant sentinel sites. Data from July 2017 to March 2019 were monitored and validated.

### Statistical analyses

Data from week 29, 2017 to week 13, 2019 were analysed. Temporal graphics were used to represent trends of indicators (in percentage or absolute number, on the y-axis) according to epidemiological weeks on the x-axis. Heat maps were used to map indicators (absolute number) according to epidemiological weeks on the x-axis and latitude of sentinel sites on the y-axis. The malaria sentinel surveillance system was described in terms of completeness and promptness of data, malaria case management, and stock shortage of inputs for malaria case management. The number of confirmed malaria cases and malaria-related deaths were shown both by target group and in each sentinel site. All statistical analyses were performed using R software version 3.5.3 [13].

## Results

### Data completeness and promptness

**Fig. 3:**
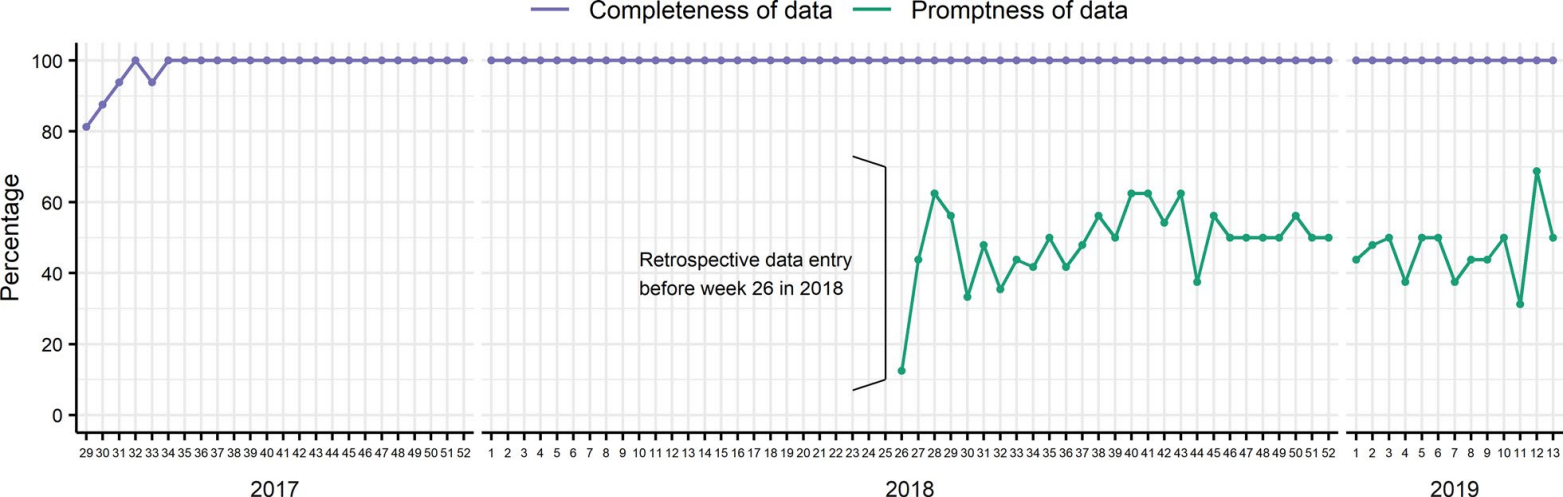
Completeness and promptness of data. Data promptness was calculated when the online data entry platform was set up in week 26, 2018

All sites provided complete data (except at the beginning of the pilot study) but not in a timely manner (Fig. 3). The median weekly promptness was 50% (interquartile range (IQR): 44–51), ranging from a minimum of 13% to a maximum of 70%. The median time to entry the form was 4 days (IQR: 4–23, minimum 1 day, maximum 275 days). The country’s target for completeness and promptness of data is 100%.

### Trends in confirmed malaria cases

**Fig. 4:**
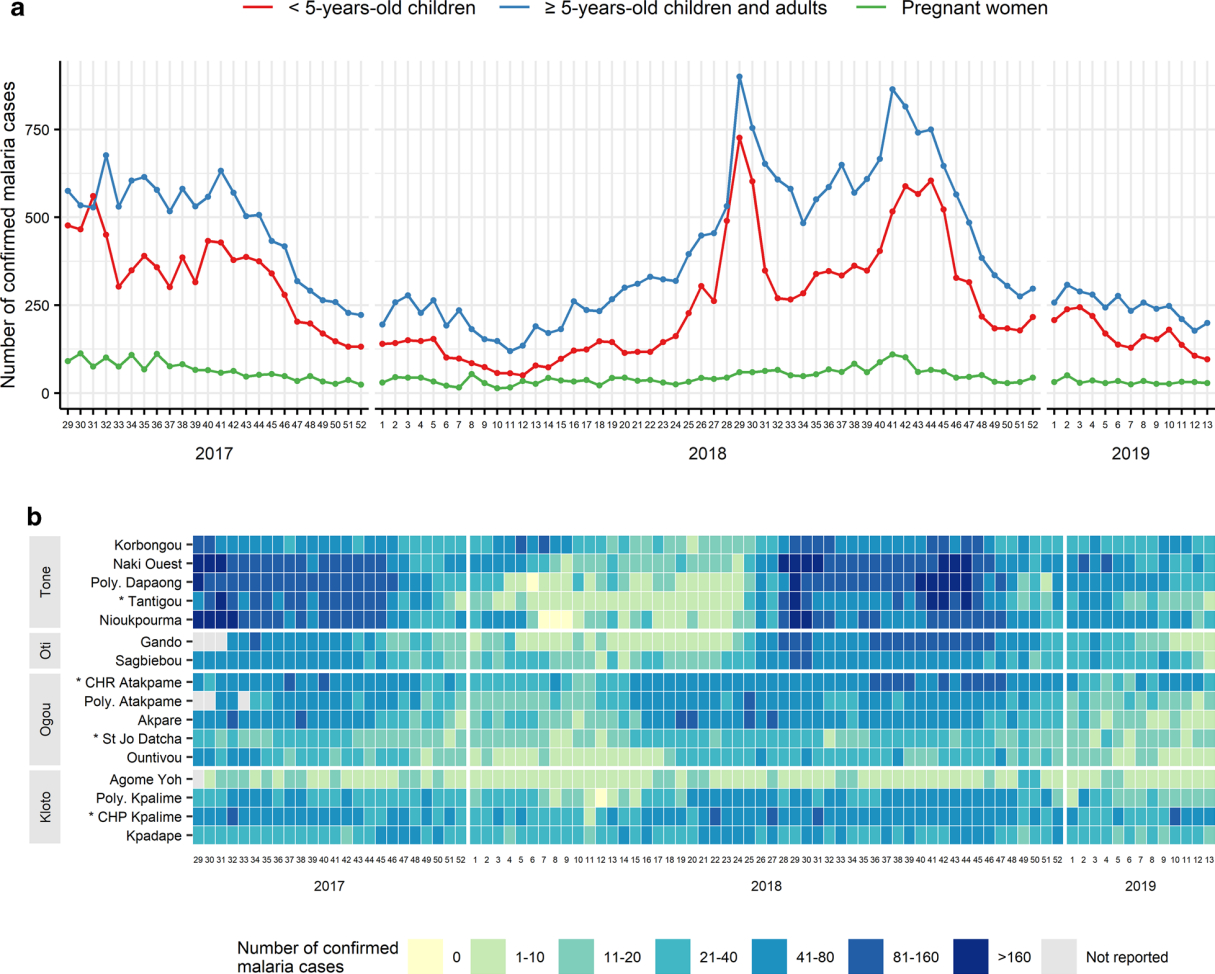
Evolution of the confirmed malaria case numbers. **a** describes the number of confirmed malaria cases by target group. **b** describes the number of confirmed malaria cases in each sentinel site. Sentinel sites marked with an asterisk are hospitals; other sentinel sites are peripheral care units. Sentinel sites were classified according to their latitude

Figure 4 shows the evolution of confirmed malaria case number by target group and by sentinel site. The number of confirmed malaria cases increased during the rainy seasons both in children under 5 years old and in children over 5 years old and adults (Fig. 4a). The mean number of confirmed malaria cases in children under 5 years old was 260.2 cases per week (standard deviation (SD): 153.4) and varied from 50 to 726 cases. The mean number of confirmed malaria cases in children over 5 years old and adults was 405.6 cases per week (SD: 192.5) and varied from 119 to 900 cases. In pregnant women, the number of confirmed malaria cases remained constant over the period. The mean number of confirmed malaria cases in pregnant women was 48.8 cases per week (SD: 23.3) and varied from 14 to 113 cases. Figure 4b describes the heat map of confirmed malaria cases in each sentinel site. The Tone and Oti districts reported more confirmed malaria cases during the rainy seasons than the Ogou and Kloto districts.

**Fig. 5:**
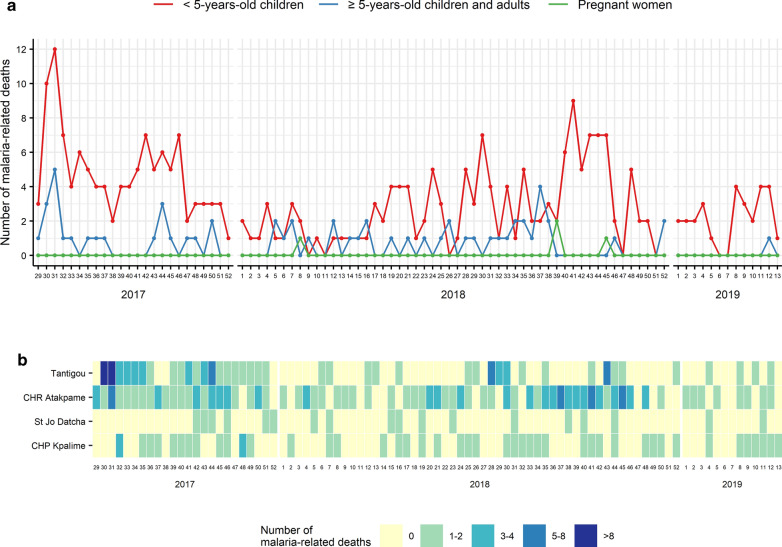
Evolution of malaria-related death numbers. **a** describes the number of malaria-related deaths by target group. **b** describes the number of malaria-related deaths in each sentinel site. Sentinel sites were classified according to their latitude

Figure 5 shows the evolution of malaria-related death number by target group and by sentinel site. Data on malaria-related deaths were only collected in hospitals. Malaria-related deaths occurred mainly in children under 5 years old and increased during the rainy seasons (Fig. 5a). The mean number of malaria-related deaths in children under 5 years old was 3.2 deaths per week (SD: 2.4) and reached 12 deaths per week. The mean number of malaria-related deaths in children over 5 years old and adults was 0.7 deaths per week (SD: 1.0) and reached 5 deaths per week. As shown in Fig. 5a, malaria-related deaths among pregnant women were a rare occurrence (0.04 deaths per week on average; SD: 0.26). Figure 5b describes the heat map of malaria-related deaths in each hospital.

### Compliance of malaria case management with national directives

**Fig. 6:**
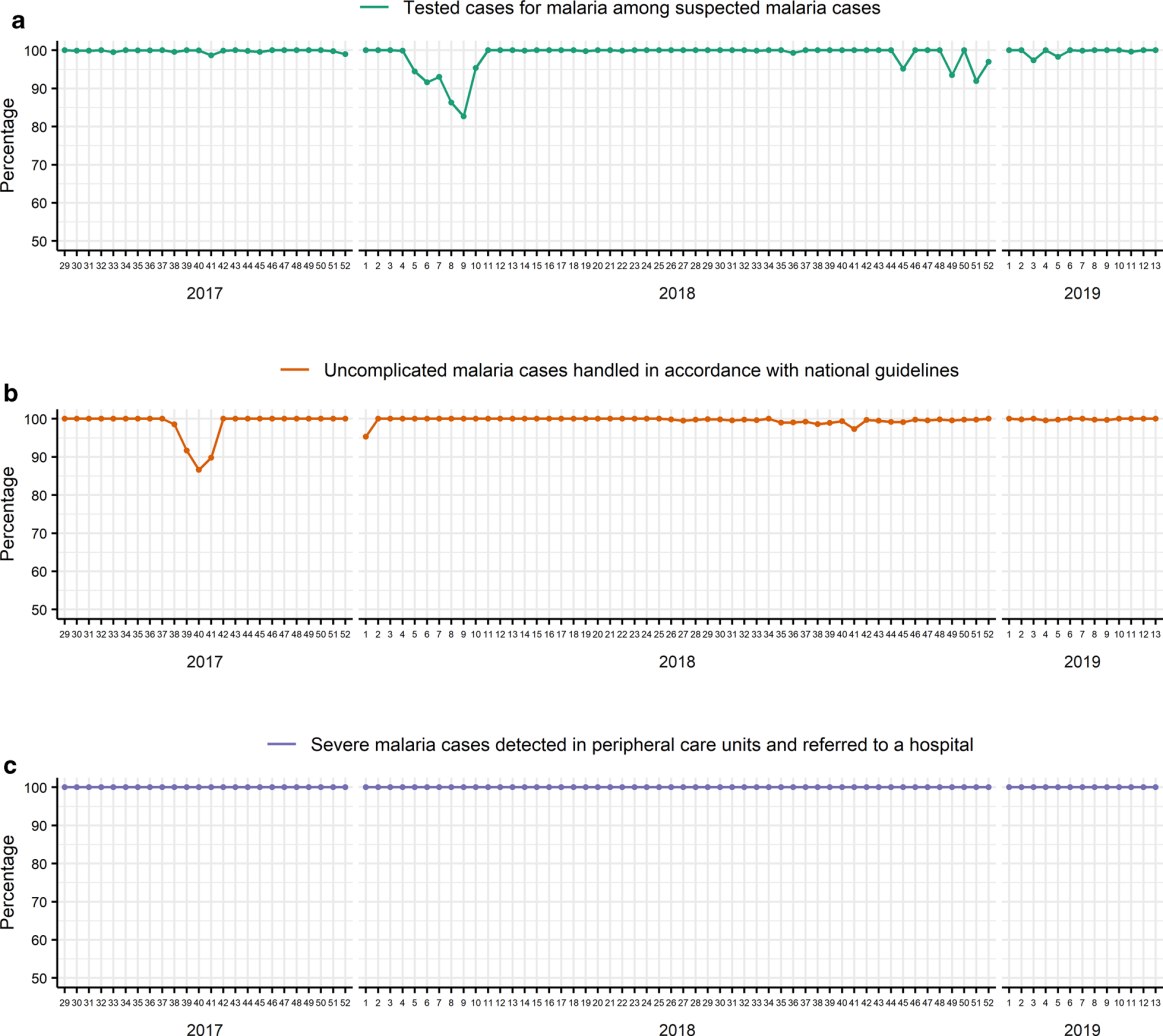
Compliance of malaria case management with national directives. Three indicators were observed, such as **a** the percentage of tested cases for malaria among suspected malaria cases, **b** the percentage of uncomplicated malaria cases handled in accordance with national guidelines, and **c** the percentage of severe malaria cases detected in peripheral care units that were referred to a hospital

The compliance of malaria case management with national directives is shown in Fig. 6. From week 29 in 2017 to week 13 in 2019, the mean percentage of tested cases for malaria among suspected malaria cases was 99.0% (SD: 2.9) (Fig. 6A). The percentage of tested cases decreased to 82.7% at the beginning of 2018 (week 5 to week 9) and fluctuated slightly at the end of 2018 (weeks 45, 49 and 51). The mean percentage of uncomplicated malaria cases handled in accordance with national guidelines was 99.4% (SD: 2.0) from week 29 in 2017 to week 13 in 2019. A decrease in this percentage was observed from week 38 to week 41 in 2017 (Fig. 6b). A slight fluctuation was observed from the second half of 2018 onwards. However, all other sites complied with national recommendations for the treatment of uncomplicated malaria cases. As shown in Fig. 6c, peripheral care units referred all severe malaria cases to a hospital during the study period analysed.

### Stock shortage of inputs for malaria case management

**Fig. 7:**
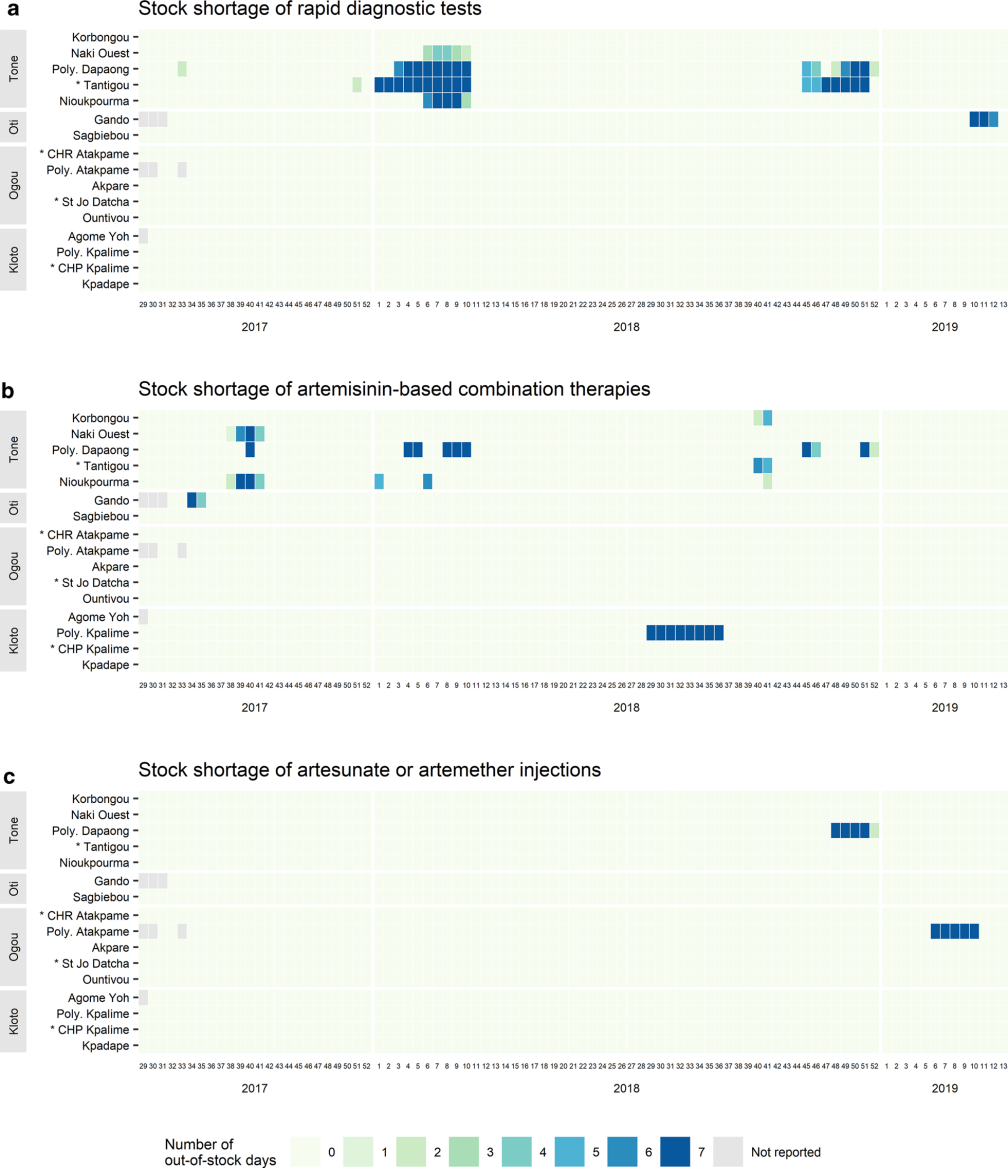
Stock shortage of rapid diagnostic tests (**a**), artemisinin-based combination therapies (**b**) and artesunate or artemether injections (**c**) in each sentinel site. Sentinel sites marked with an asterisk are hospitals; other sentinel sites are peripheral care units. Sentinel sites were classified according to their latitude

Figure 7 shows the stock shortage of RDTs, ACT, injectable artesunate or artemether, used to manage malaria cases in sentinel sites. RDTs were out of stock in 4 sentinel sites at the beginning of 2018 and in 2 sentinel sites at the end of 2018 (Fig. 7a). All these sites were in the Tone district. The longest consecutive RDT stock shortage in a sentinel site was 10 weeks. Stock shortage was occurring for ACT, mostly in the Savanes region (Fig. 7b). The site that had the longest consecutive ACT stock shortage (8 weeks in 2018) was in the Plateaux region. Figure 7c shows that no hospital was out of stock of injectable artesunate or injectable artemether. Peripheral care units did not manage severe malaria cases but had a stock of injectable artemether to use when severe malaria cases were referred. Two peripheral care units were out of stock during the study period analysed.

## Discussion

Togo uses a combination of strategies and interventions in order to control malaria. Malaria sentinel surveillance is a new tool, in pilot phase, implemented in July 2017. Only a few West African countries, such as Senegal, have experimented it. On the one hand, following evolution of malaria cases in real time enables the National Malaria Control Programme of Togo to take active and appropriate measures based on the collected data and to improve decision-making for the malaria response. On the other hand, retrospective analyses of sentinel surveillance data would make it possible to assess the effectiveness of actions in the field and to adapt or correct them if necessary.

Since July 2017, Togo has set up a malaria sentinel surveillance system in 16 heath facilities that provide weekly data on malaria morbidity and mortality. The first observation is that this sentinel surveillance system is operational and data completeness was excellent during the first 20 months of data collection. This surveillance system showed good performance in the management of malaria cases. The mean percentages of tested cases for malaria among suspected malaria cases, of uncomplicated malaria cases handled in accordance with national guidelines and of severe malaria cases detected in peripheral care units that were referred to a hospital were over 99%. Nevertheless, occasional fluctuations were observed (Fig. 6). At the beginning of 2018, 4 of the 5 sentinel sites in the Tone district were out of stock of RDTs (Fig. 7a), which explains the decrease in tested cases among suspected cases (Fig. 6a). Malaria diagnosis by microscopy was not available in all sentinel sites. From week 38 to week 41 in 2017, one sentinel site (Nioukpourma) was out of stock of ACT and treated all confirmed malaria cases with quinine (Fig. 7b), leading to a decrease in the percentage of uncomplicated malaria cases handled in accordance with national guidelines (Fig. 6b). The slight fluctuation observed from the second half of 2018 onwards (Fig. 6b) was due to a sentinel site (Gando) located in a rural area and remote from a hospital that treated several uncomplicated malaria cases as severe malaria cases. Malaria case management is a key component of malaria surveillance to produce high quality data and to give an accurate picture of malaria in order to make decisions in malaria control activities. However, poor malaria case management should not be an impediment to the establishment of a surveillance system. Indeed, the implementation of a surveillance system helps to improve and maintain good malaria case management, as shown in a study in Uganda by Sserwanga et al. In this study, the authors compared the first 3 months (at the end of 2006) with the last 3 months (at the beginning of 2010) of surveillance. Their results showed an increase in the percentage of tested patients among patients with suspected malaria (from 39 to 97%, p < 0.001) and an increase in the percentage of patients with an appropriate decision to prescribe anti-malarial therapy (from 64 to 95%, p < 0.001) [14]. In this paper, the results showed that the number of confirmed malaria cases increased during the rainy seasons, both in children under 5 years old and in children over 5 years old and adults. Similar trends were observed with national routine data [3]. This result is consistent because the climatic conditions during the rainy seasons in Togo are favourable to the proliferation of mosquitoes and therefore to the increase of malaria cases. This study also showed that malaria-related deaths occurred mainly in children under 5 years old, as reported in the 2018 report of the National Malaria Control Programme [3] and the previous reports. Overall, the sentinel surveillance system provided similar epidemiological information that the routine monitoring system. Regarding reporting and feedback mechanisms, only hierarchical mechanism was initially foreseen. The use of a cross-platform mobile application was necessary to meet the need for rapid information sharing. Both mechanisms were used and complemented each other.

This sentinel surveillance system needs to be improved in order to achieve its primary objective. Firstly, supply should not be disrupted in the sentinel sites and should be exemplary in malaria case management from diagnosis to treatment. Secondly, promptness of data is a key component of a dynamic surveillance system [15]. Data were available in time on the paper forms but were entered with delay on the online platform. Although the sentinel sites received money on their mobile phone to connect to the internet, not all sites had a good network, especially in rural areas. Some sentinel site managers had to go to the nearest city to get internet access. Some enhancements to the data entry platform may be possible to overcome this problem, such as offline data entry and synchronization of data according to availability of the internet. Other countries have used mobile phone technology to improve data timeliness [16, 17]. Data visualisation by health facility staff, as well as at all levels of the malaria sentinel surveillance system, is one way to improve the timeliness of data, as shown by Chisha et al*.* in Zambia. Clinic staff had the ability to examine malaria trends in their clinic or in other clinics through a dashboard available on an online platform [18]. In Togo, data are described graphically on the online data entry platform, but it seems to be little used and the proposed graphics need to be improved. Thirdly, data monitoring should be carried out on a regular basis. Written and standardized procedures in data monitoring are required, including inspection visits to sentinel sites. Fourth, the surveillance system must continue to provide data despite the turnover of health workers. Training and retraining of site managers is a key point to ensure the availability of quality data in a timely manner. The evaluation of the malaria surveillance system in Kano State, Nigeria, showed that data quality was improved through integrated supportive supervision and on-the-job training [19]. Fifth, the sample of sentinel sites must be such as to ensure that the data collected are nationally representative. The inclusion of new health facilities will need to take this into account. Priority will be given to public health facilities or private health facilities that are accustomed to working with the National Malaria Control Programme in order to ensure data reporting. The integration of private sector into surveillance systems is a major issue in achieving adequate surveillance coverage [20]. However, it is identified as one of the main gaps in the assessment of surveillance systems [21]. Finally, only 2 attributes of surveillance system were assessed in this article, namely completeness and promptness of data. A thorough evaluation would be beneficial to improve this malaria sentinel surveillance system. For example, the CDC Updated Guidelines for evaluating public health surveillance systems suggested other indicators such as usefulness, simplicity, flexibility, acceptability, sensitivity, predictive value positive, representativeness and stability [22].

This is the first study since the implementation of the sentinel surveillance intervention in Togo. Articles describing the establishment of malaria surveillance systems in sub-Saharan African countries are scarce in the literature. A study published in 2014 by Yukich et al. described the implementation of a malaria sentinel surveillance system in 10 sentinel sites in Oromia Regional State, Ethiopia and discussed lessons learned [17]. The challenges identified are similar to those observed in this article such as the timeliness of data and the generalization of the system to achieve the objectives of reducing malaria morbidity and mortality in the country. This intervention is not sufficiently widespread to provide clear impact in malaria-endemic countries. Many countries have a malaria surveillance system, similar to the routine data collection system in Togo, but they do not make sufficient use of the data collected to make decisions [21]. Togo is one of the few West African countries to experiment this approach, and, as well as other West African countries, needs to be encouraged and supported by technical and financial partners.

## Conclusions

Surveillance is one of the essential pillars recommended by the WHO to all countries affected by malaria. In Togo, the malaria surveillance system, through the results of this study, is promising and can become a decision support tool to guide the malaria response. For the moment, it essentially allows real-time notification of malaria cases in the sentinel sites. Efforts are needed to make it more dynamic and efficient and avoid it becoming a duplication of the routine data collection system in place in the country for decades. This new system needs to be strengthened to bring real gain to the fight against malaria in Togo.

## Data Availability

Not applicable.

## Abbreviations

ACT: Artemisinin-based combination therapies
IQR: Interquartile range
RDT: Rapid diagnostic test
SD: Standard deviation
WHO: World Health Organization
95% CI: 95% Confidence interval
